# Crystal structures and Hirshfeld surface analyses of 2-amino-4-(4-bromo­phen­yl)-6-oxo-1-phenyl-1,4,5,6-tetra­hydro­pyridine-3-carbo­nitrile hemi­hydrate and 1,6-di­amino-2-oxo-4-phenyl-1,2-di­hydro­pyridine-3,5-dicarbo­nitrile

**DOI:** 10.1107/S2056989022007356

**Published:** 2022-07-26

**Authors:** Farid N. Naghiyev, Victor N. Khrustalev, Nikolai U. Venskovsky, Tatiana A. Tereshina, Ali N. Khalilov, Mehmet Akkurt, Ajaya Bhattarai, İbrahim G. Mamedov

**Affiliations:** a Department of Chemistry, Baku State University, Z. Khalilov str. 23, Az, 1148, Baku, Azerbaijan; b Peoples’ Friendship University of Russia (RUDN University), Miklukho-Maklay St.6, Moscow, 117198, Russian Federation; c N. D. Zelinsky Institute of Organic Chemistry RAS, Leninsky Prosp. 47, Moscow, 119991, Russian Federation; d "Composite Materials" Scientific Research Center, Azerbaijan State Economic University (UNEC), H. Aliyev str. 135, Az 1063, Baku, Azerbaijan; e Department of Physics, Faculty of Sciences, Erciyes University, 38039 Kayseri, Turkey; f Department of Chemistry, M.M.A.M.C (Tribhuvan University) Biratnagar, Nepal; University of Neuchâtel, Switzerland

**Keywords:** crystal structure, disorder, hydrogen bonds, dimers, van der Waals inter­actions, Hirshfeld surface analysis

## Abstract

In 2-amino-4-(4-bromo­phen­yl)-6-oxo-1-phenyl-1,4,5,6-tetra­hydro­pyridine-3-carbo­nitrile hemihydrate, C_18_H_14_BrN_3_O·0.5H_2_O, (I), pairs of mol­ecules are linked by pairs of N—H⋯N hydrogen bonds, forming dimers with an *R* ^2^_2_(12) ring motif. The dimers are connected by N—H⋯Br and O—H⋯O hydrogen bonds, and C—Br⋯π inter­actions, forming layers parallel to the (010) plane. 1,6-Di­amino-2-oxo-4-phenyl-1,2-di­hydro­pyridine-3,5-dicarbo­nitrile, C_13_H_9_N_5_O, (II), crystallizes in the triclinic space group *P*  with two independent mol­ecules (IIA and IIB) in the asymmetric unit. In the crystal of (II), mol­ecules IIA and IIB are linked by inter­molecular N—H⋯N and N—H⋯O hydrogen bonds into layers parallel to (001). These layers are connected along the *c-*axis direction by weak C—H⋯N contacts. C—H⋯π and C—N⋯π inter­actions connect adjacent mol­ecules, forming chains along the *a-*axis direction. In (I) and (II), the stability of the packing is ensured by van der Waals inter­actions between the layers. In (I), Hirshfeld surface analysis showed that the most important contributions to the crystal packing are from H⋯H (37.9%), C⋯H/H⋯C (18.4%), Br⋯H/H⋯Br (13.3%), N⋯H/H⋯N (11.5%) and O⋯H/H⋯O (10.0%) inter­actions, while in (II), H⋯H inter­actions are the most significant contributors to the crystal packing (27.6% for mol­ecule IIA and 23.1% for mol­ecule IIB).

## Abstract 2

In 2-amino-4-(4-bromo­phen­yl)-6-oxo-1-phenyl-1,4,5,6-tetra­hydro­pyridine-3-carbo­nitrile hemihydrate, pairs of mol­ecules of are joined by pairs of N—H⋯N hydrogen bonds, producing a dimer with an 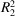 (12) ring pattern. Further N—H⋯Br and O—H⋯O hydrogen bonds, as well as C—Br⋯π inter­actions, link the dimers, producing layers parallel to the (010) plane. In the crystal of 1,6-di­amino-2-oxo-4-phenyl-1,2-di­hydro­pyridine-3,5-dicarbo­nitrile, mol­ecules are joined into layers parallel to (001) *via* inter­molecular N—H⋯N and N—H⋯O hydrogen bonds. These layers are joined along the *c* axis by weak C—H⋯N contacts. Additionally, C—H⋯π and C—N⋯π inter­actions link nearby mol­ecules, producing chains along the *a* axis.

## 1. Chemical context

The formation of C—C, C—O, and C—N bonds is one of the essential transformation reactions of organic chemistry (Zubkov *et al.*, 2018 ▸; Shikhaliyev *et al.*, 2019 ▸; Viswanathan *et al.*, 2019 ▸; Gurbanov *et al.*, 2020 ▸). Nitro­gen-containing heterocycles, especially tetra­hydro­pyridine homologs, are well-known heterocyclic scaffolds that exhibit a broad spectrum of biological and pharmaceutical activities (Sośnicki & Idzik, 2019 ▸; Sangwan *et al.*, 2022 ▸). Being an important structural fragment of various natural products, they play a key role in cell metabolism. In view of the growing biological value of pyridine derivatives, we have considered the study of this class of compounds (Naghiyev *et al.*, 2020*b* ▸) to be of great inter­est. Thus, in the framework of our ongoing structural studies (Naghiyev *et al.*, 2020*a* ▸,*b* ▸, 2021 ▸, 2021*a* ▸,*b* ▸, 2022 ▸; Khalilov *et al.*, 2022 ▸), we report here the crystal structures and Hirshfeld surface analyses of 2-amino-4-(4-bromo­phen­yl)-6-oxo-1-phenyl-1,4,5,6-tetra­hydro­pyridine-3-carbo­nitrile hemihydrate (I) and 1,6-di­amino-2-oxo-4-phenyl-1,2-di­hydro­pyridine-3,5-dicarbo­nitrile (II). 
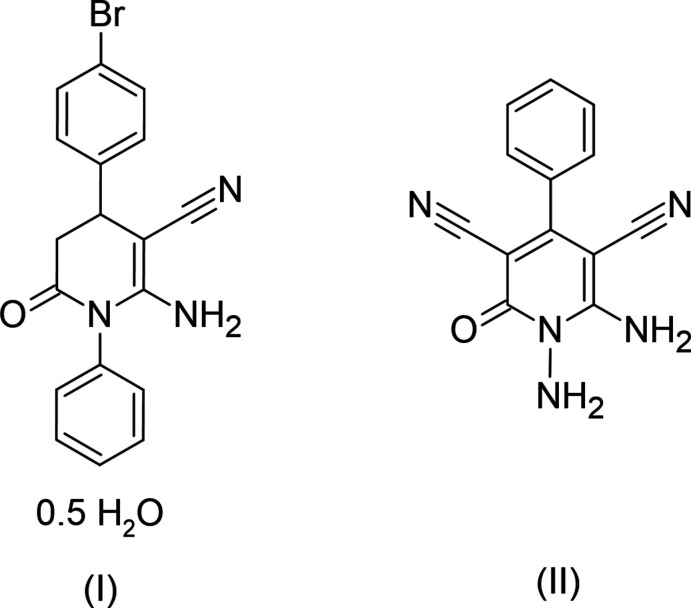


## 2. Structural commentary

**Figure 1:**
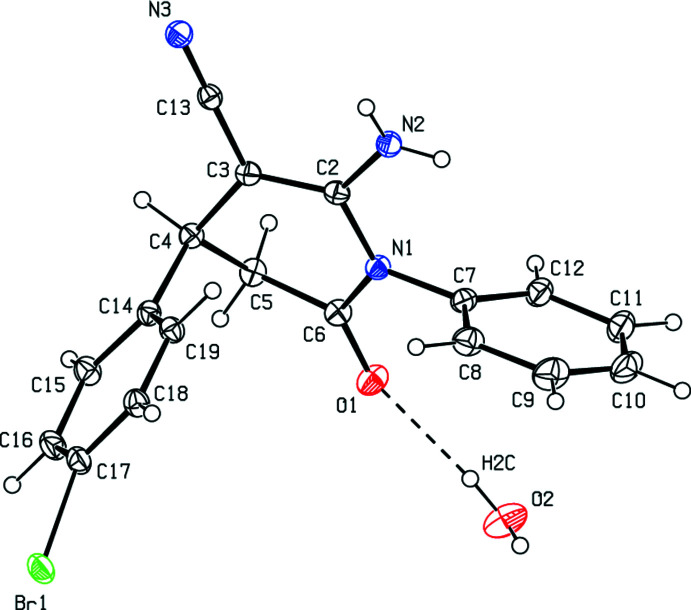
The mol­ecular structure of compound (I) with displacement ellipsoids drawn at the 30% probability level. The O—H⋯O hydrogen bond is drawn with a dashed line. Only the major component of the bromide disorder is shown for clarity.

Compound (I) crystallizes in the monoclinic space group *C*2/*c* with *Z* = 4. In (I) (Fig. 1 ▸), the conformation of the central di­hydro­pyridine ring is close to screw-boat with puckering parameters (Cremer & Pople, 1975 ▸) *Q*_T_ = 0.4650 (16) Å, θ = 61.3 (2)° and φ = 211.4 (2)°. The phenyl (C7–C12) and bromo­phenyl (C14–C19) rings form dihedral angles of 64.68 (8) and 88.25 (7)°, respectively, with the mean plane of the central di­hydro­pyridine ring. The chirality about the C4 atom is *S* for this molecule, but both enantiomers are present in the crystal. The Br atom is disordered over two sites in a 0.59 (2):0.41 (2) ratio.

**Figure 2:**
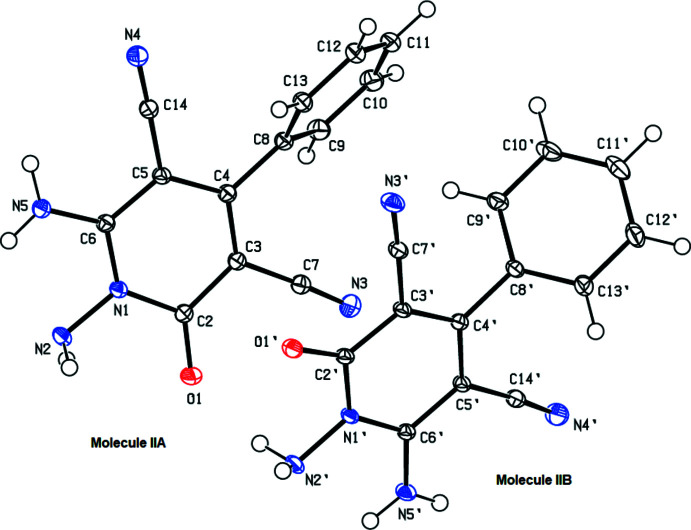
The mol­ecular structure of compound (II). Displacement ellipsoids are drawn at the 50% probability level.

**Figure 3:**
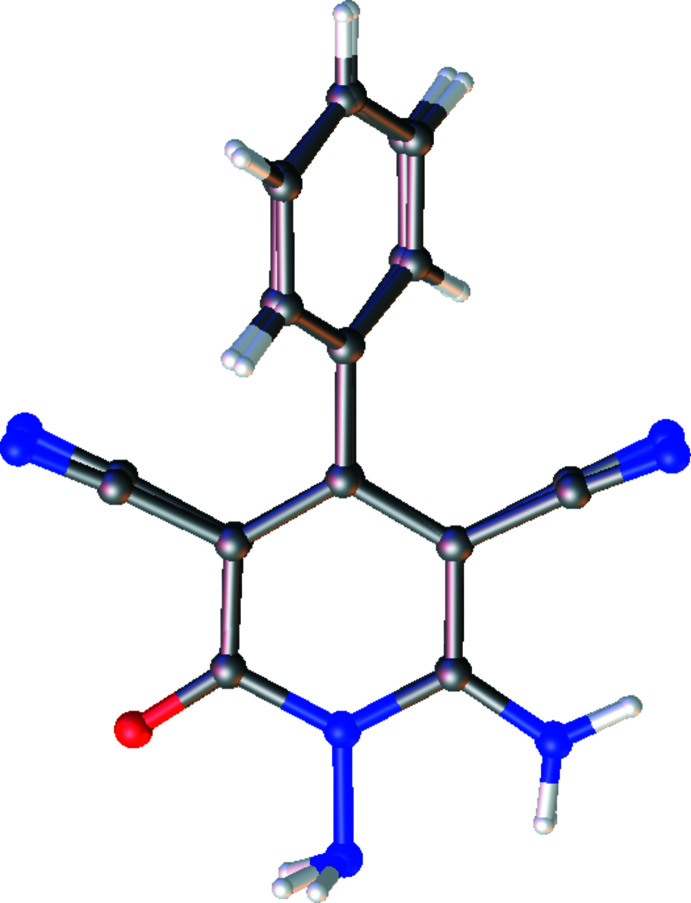
Overlay image of the two independent mol­ecules (IIA and IIB) in the asymmetric unit of compound (II). Color code: carbon (gray), hydrogen (white), nitro­gen (blue) and oxygen (red).

Compound (II) (Fig. 2 ▸) contains two independent mol­ecules (IIA and IIB, atom labels for mol­ecule IIB including the suffix ’) in the asymmetric unit. Fig. 3 ▸ shows the overlay of mol­ecules IIA and IIB (r.m.s. deviation = 0.210 Å). The pyridine and phenyl rings subtend dihedral angles of 52.95 (4)° in mol­ecule IIA and 56.75 (3) ° in mol­ecule IIB.

The geometric parameters of mol­ecules (I), (IIA) and (IIB) are normal and comparable to those of related compounds listed in the *Database survey* section.

## 3. Supra­molecular features

**Figure 4:**
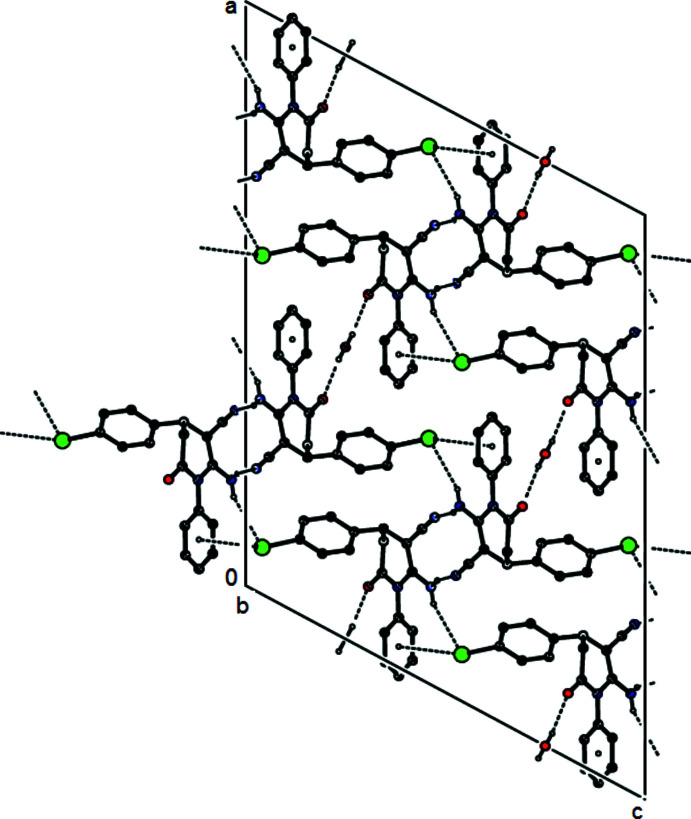
Crystal packing of compound (I) viewed down the *b* axis, showing the O—H⋯O, N—H⋯O and C—Br⋯π inter­actions.

In (I), pairs of N—H⋯N hydrogen bonds connect the mol­ecules, forming dimers with an 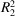 (12) ring motif (Fig. 4 ▸, Table 1 ▸). Further N—H⋯Br and O—H⋯O hydrogen bonds, as well as C—Br⋯π inter­actions [C17—Br1⋯*Cg*2^vii^: Br1⋯*Cg*2^vii^ = 3.493 (2) Å, C17⋯*Cg*2^vii^ = 5.3027 (18) Å, C17—Br1⋯*Cg*2^vii^ = 157.80 (14)°; C17—Br1*A*⋯*Cg*2^vii^: Br1*A*⋯*Cg*2^vii^ = 3.434 (6) Å, C17⋯*Cg*2^vii^ = 5.3027 (18) Å, C17—Br1*A*⋯*Cg*2^vii^ = 164.8 (3)°; symmetry code: (vii) *x*, 2 − *y*, −*z* + 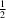 ; *Cg*2 is the centroid of the C7–C12 phenyl ring], link the dimers, forming layers parallel to the (010) plane (Fig. 4 ▸). Inter­layer van der Waals inter­actions strengthen the mol­ecular packing.

**Figure 5:**
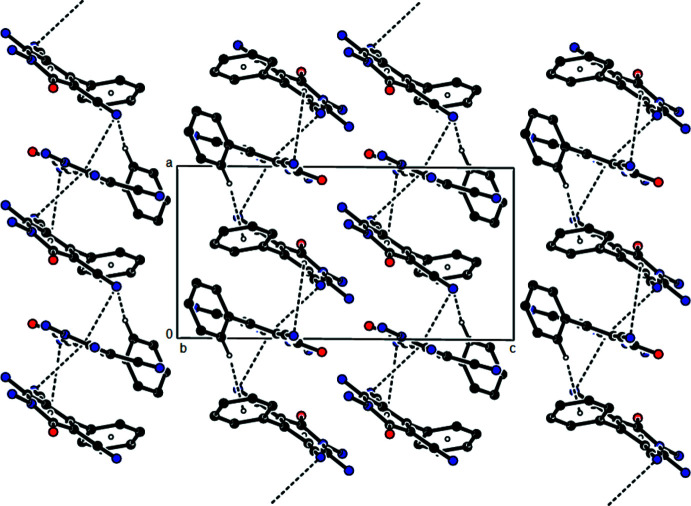
View down the *b* axis of compound (II) showing the C—H⋯π and C—N⋯π hydrogen bonds (dashed lines). The intra­molecular C—N⋯π inter­action in mol­ecule IIA is omitted for clarity.

In the crystal of (II), mol­ecules IIA and IIB are linked by inter­molecular N—H⋯N and N—H⋯O hydrogen bonds (Table 2 ▸) into layers parallel to (001). These layers are connected along the *c*-axis direction by weak C—H⋯N contacts. Furthermore, C—H⋯π (Table 1 ▸) and C—N⋯π [C7—N3⋯*Cg*3: N3⋯*Cg*3 = 3.0831 (8) Å, C7⋯*Cg*3 = 3.3390 (8) Å, C7—N3⋯*Cg*3 = 92.50 (5)°; C7′—N3′⋯*Cg*1^v^: N3′⋯*Cg*1^v^ = 3.4626 (9) Å, C7′⋯*Cg*1^v^ = 3.7591 (9) Å, C7′—N3′⋯*Cg*1^v^ = 95.78 (6)°; C14—N4⋯*Cg*3^vi^: N4⋯*Cg*3^vi^ = 3.3807 (7) Å, C14⋯*Cg*3^vi^ = 3.8513 (7) Å, C14—N4⋯*Cg*3^vi^ = 105.23 (5)°; symmetry codes: (v) 1 + *x*, *y*, *z*, (vi) −1 + *x*, 1 + *y*, *z*; where *Cg*1 and *Cg*3 are the centroids of the N1/C2 –C6 and N1′/C2′ –C6′ pyridine rings of mol­ecules IIA and IIB, respectively] inter­actions connect the adjacent mol­ecules, forming chains along the *a*-axis direction (Fig. 5 ▸). The stability of the mol­ecular packaging is ensured by van der Waals inter­actions between the layers.

## 4. Hirshfeld surface analyses

**Figure 6:**
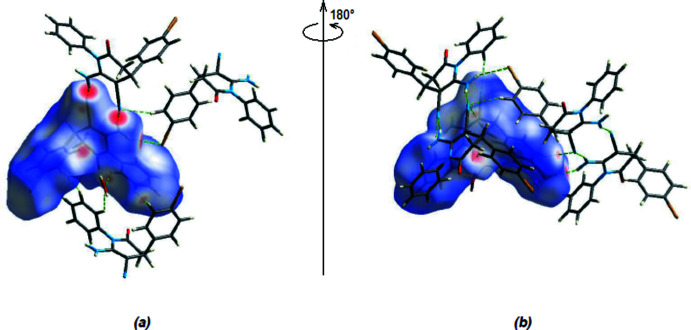
(*a*) Front and (*b*) back views of the Hirshfeld surfaces mapped over *d*_norm_ for (I).

*CrystalExplorer17.5* (Turner *et al.*, 2017 ▸) was used to construct Hirshfeld surfaces and generate the related two-dimensional fingerprint plots to illustrate the inter­molecular inter­actions for mol­ecules (I) and (II). The *d*_norm_ mappings of (I) were conducted in the range −0.4915 to +1.2143 a.u. Bright-red circles on the *d*_norm_ surfaces (Fig. 6 ▸ *a*,*b*) represent N—H⋯O and O—H⋯O inter­action zones. Red areas on the Hirshfeld surfaces are also caused by the N—H⋯Br and C—H⋯N inter­actions (Tables 1 ▸ and 3 ▸).

**Figure 7:**
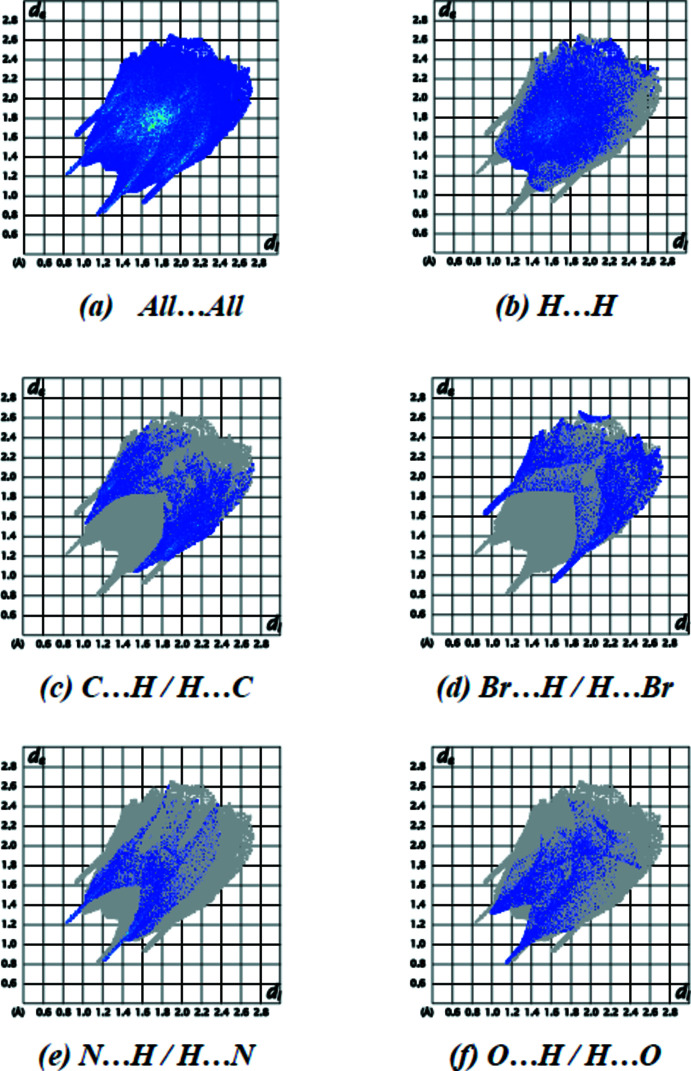
The two-dimensional fingerprint plots of (I), showing all inter­actions (*a*), and those delineated into H⋯H (*b*), C⋯H/H⋯C (*c*), Br⋯H/H⋯Br (*d*), N⋯H/H⋯N (*e*) and O⋯H/H⋯O (*f*) inter­actions. The *d*_i_ and *d*_e_ values are the closest inter­nal and external distances (in Å) from given points on the Hirshfeld surfaces.

The fingerprint plots of (I) (Fig. 7 ▸) show that, while H⋯H (37.9%; Fig. 7 ▸ *b*) inter­actions provide the highest contribution (Table 3 ▸), as would be expected for a mol­ecule with so many H atoms, C⋯H/H⋯C (18.4%; Fig. 7 ▸ *c*), Br⋯H/H⋯Br (13.3%; Fig. 7 ▸ *d*), N⋯H/H⋯N (11.5%; Fig. 7 ▸ *e*) and O⋯H/H⋯O (10.0%; Fig. 7 ▸ *f*) contacts are also significant. Table 5 ▸ shows the contributions of all contacts.

**Figure 8:**
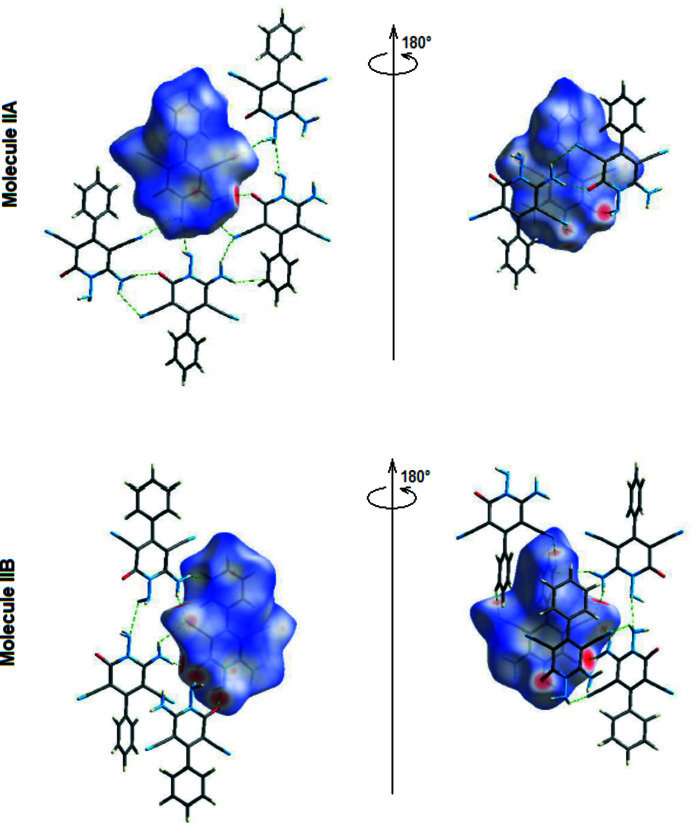
Front and back views of the three-dimensional Hirshfeld surface of mol­ecules (IIA) and (IIB) plotted over *d*_norm_ in the range −0.5399 to 1.2085 a.u. for (IIA) and in the range −0.5388 to 1.1921 a.u. for (IIB).

In (II), the *d*_norm_ mappings for mol­ecules IIA and IIB were performed in the ranges −0.5399 to 1.2085 a.u. and −0.5388 to 1.1921 a.u., respectively. The locations of N—H⋯N inter­actions are shown by bright red circles on the *d*_norm_ surfaces (Fig. 8 ▸ *a*,*b* for A and Fig. 8 ▸ *c*,*d* for B). Red spots on the Hirshfeld surfaces are also caused by N—H⋯O inter­actions (Tables 2 ▸ and 4 ▸).

**Figure 9:**
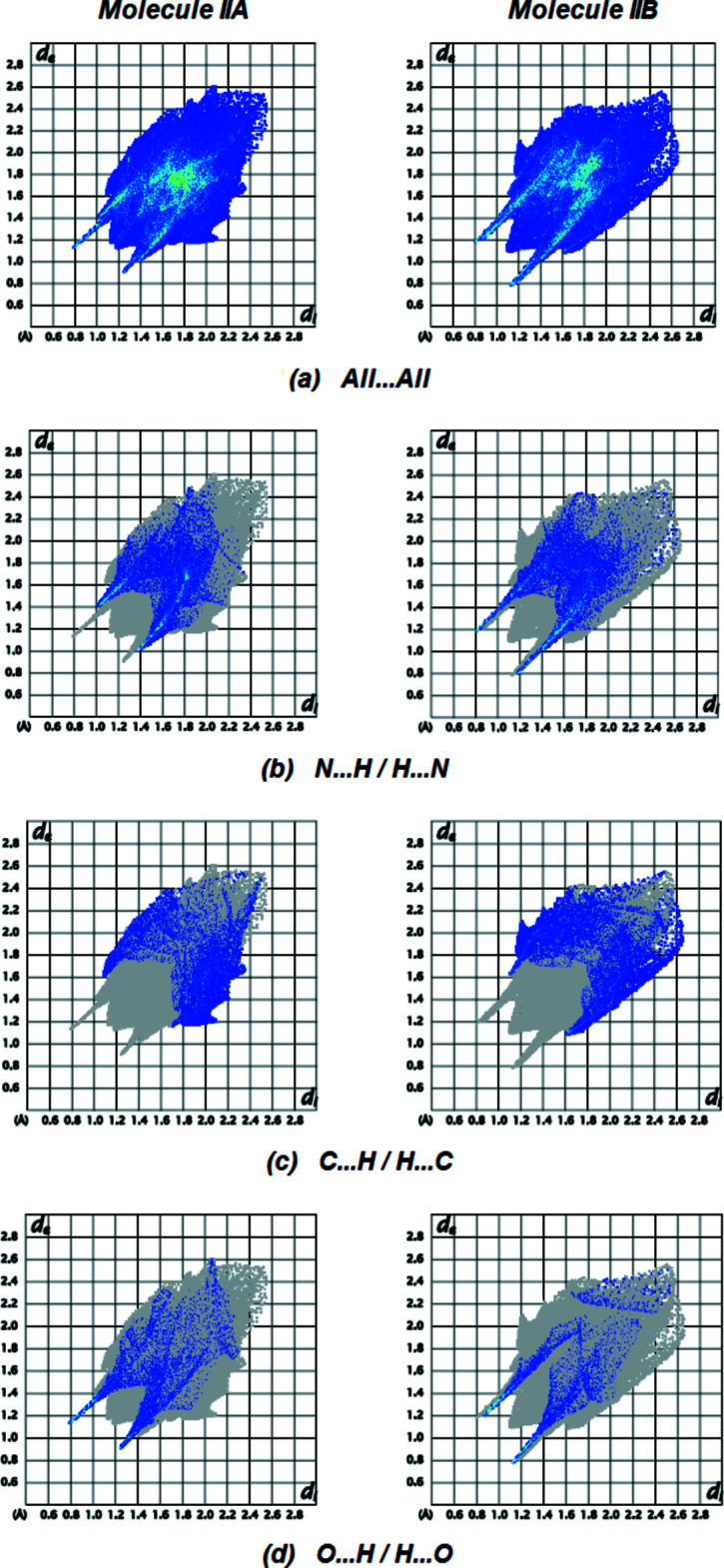
The full two-dimensional fingerprint plots for mol­ecules (IIA) and (IIB), showing all inter­actions (*a*) and those delineated into N⋯H/H⋯N (*b*), C⋯H/H⋯C (*c*) and O⋯H/H⋯O (*d*) inter­actions. The *d*_i_ and *d*_e_ values are the closest inter­nal and external distances (in Å) from given points on the Hirshfeld surface.

Fig. 9 ▸ displays the full two-dimensional fingerprint plot and those delineated into the major contacts. H⋯H inter­actions (Fig. 9 ▸ *b*; 27.6% contribution for IIA; 23.1% for IIB) are the major factor in the crystal packing with N⋯H/H⋯N (Fig. 9 ▸ *c*; 25.2% for IIA; 28.3% for IIB), C⋯H/H⋯C (Fig. 9 ▸ *d*; 15.2% for IIA; 21.2% for IIB) and O⋯H/H⋯O (Fig. 9 ▸ *e*; 11.4% for IIA; 8.8% for IIB) inter­actions representing the next highest contributions. The percentage contributions of comparative weaker inter­actions of mol­ecules IIA and IIB are given in Table 6 ▸. The surroundings of mol­ecules IIA and IIB are quite similar, as seen by the data comparison.

## 5. Database survey

A search of the Cambridge Structural Database (CSD, Version 5.42, update of September 2021; Groom *et al.*, 2016 ▸) gave eleven compounds closely related to the title compounds, *viz.* CSD refcodes YAXQAT (**I**) (Mamedov *et al.*, 2022 ▸), OZAKOS (**II**) (Naghiyev *et al.*, 2021 ▸), JEBREQ (**III**) (Mohana *et al.*, 2017 ▸), JEBRAM (**IV**) (Mohana *et al.*, 2017 ▸), SETWUK (**V**) (Suresh *et al.*, 2007 ▸), SETWOE (**VI**) (Suresh *et al.*, 2007 ▸), IQEFOC (**VII**) (Naghiyev *et al.*, 2021*a* ▸), MOKBUL (**VIII**) (Mohamed *et al.*, 2014 ▸), PAVQIO (**IX**) (Al-Said *et al.*, 2012 ▸), YIZGOE01 (**X**) (Jia & Tu, 2008 ▸) and YIBZAL (**XI**) (Eyduran *et al.*, 2007 ▸).

In the crystal of (**I**) (space group: *Pc*), the two mol­ecules in the asymmetric unit are joined together by N—H⋯O hydrogen bonds, forming a dimer with an 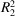 (16) ring motif. N—H⋯O and N—H⋯N hydrogen bonds link the dimers, generating chains along the *c*-axis direction, which are connected by C—Br⋯π inter­actions. In (**II**) (space group: *Pc*), inter­molecular N—H⋯N and C—H⋯N hydrogen bonds, as well as N—H⋯π and C—H⋯π inter­actions, connect mol­ecules in the crystal, generating a 3D network. In both (**III**) (space group: *P* 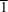 ) and (**IV**) (space group: *P* 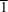 ), a supra­molecular homosynthon [ 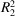 (8) ring motif] is formed through N—H⋯N hydrogen bonds. The mol­ecular structures are further stabilized by π–π stacking, and C=O⋯π, C—H⋯O and C—H⋯Cl inter­actions. In (**V**) (space group: *P*2_1_/*n*), the crystal structure is stabilized by inter­molecular C—H⋯F and C—H⋯π inter­actions, and in (**VI**) (space group: *P*2_1_/*c*), by inter­molecular C—H⋯O and C—H⋯π inter­actions. In (**VII**) (space group: *C*c), inter­molecular N—H⋯N and C—H⋯N hydrogen bonds form mol­ecular sheets parallel to the (110) and (110) planes, crossing each other. Adjacent mol­ecules are further linked by C—H⋯π inter­actions, which form zigzag chains propagating parallel to [100]. The compound (**VIII**) (space group: *P*ca2_1_) crystallizes with two independent mol­ecules, *A* and *B*, in the asymmetric unit. In the crystal, mol­ecules *A* and *B* are linked by N—H⋯S, N—H⋯N and C—H⋯S hydrogen bonds, forming a three-dimensional network. In (**IX**) (space group: *P*2_1_/c), mol­ecules are linked into a chain along the *b-*axis direction *via* C—H⋯O inter­actions. In (**X**) (space group: *P* 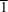 ), the crystal packing is stabilized by inter­molecular N—H⋯N, O—H⋯O and N—H⋯O hydrogen bonds. In (**XI**) (space group: *P*2_1_/c), the mol­ecules form centrosymmetric dimers *via* N—H⋯S hydrogen bonds.

## 6. Synthesis and crystallization

Compounds (I) and (II) were synthesized using reported procedures [Mamedov *et al.* (2020 ▸) and Soto *et al.* (1981 ▸), respectively]. Colorless crystals of (I) were obtained at room temperature upon slow evaporation of a homogeneous methanol solution, while colorless needle-like crystals of (II) were obtained at room temperature upon slow evaporation from an ethanol/water (3:1) homogeneous solution.

## 7. Refinement

Crystal data, data collection and structure refinement details are summarized in Table 7 ▸. In (I), the H atoms were placed at calculated positions (C—H = 0.95–1.00 Å) and refined as riding with *U*_iso_(H) = 1.2*U*_eq_(C) or 1.5*U*_eq_(C-meth­yl). The N-bound H atoms and the H atoms of the water mol­ecule located at the coordinates (0.5, *y*, 0.25) were found in a difference-Fourier map, and refined freely [N2—H2*A* = 0.82 (2), N2—H2*B* = 0.84 (2) Å, and O2—H2*C* = 0.849 (10), O2—H2*C*(−*x* + 1, *y*, −*z* + 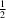 ) = 0.849 (10) Å, with *U*_iso_(H) = 1.2 or 1.5*U*_eq_(N, O). The DFIX instruction was applied to constrain the distance O2—H2*C*. The Br1 atom is disordered over two positions with refined occupancies of 0.59 (2) and 0.41 (2).

In (II), the H atoms were placed at calculated positions (C—H = 0.95 Å) and refined using a riding model with *U*_iso_(H) = 1.2*U*_eq_(C). N-bound H atoms were found in a difference Fourier map and refined freely [N2—H2*A* = 0.913 (13), N2—H2*B* = 0.916 (14), N5—H5*A* = 0.897 (14) and N5—H5*B* = 0.930 (14) Å for mol­ecule IIA, and N2′—H2*A*′ = 0.898 (13), N2′—H2*B*′ = 0.902 (15), N5′—H5*A*′ = 0.887 (15) and N5′—H5*B*′ = 0.889 (12) Å for mol­ecule IIB].

## Supplementary Material

Crystal structure: contains datablock(s) I, II, global. DOI: 10.1107/S2056989022007356/tx2055sup1.cif

Structure factors: contains datablock(s) I. DOI: 10.1107/S2056989022007356/tx2055Isup2.hkl

Supporting information file. DOI: 10.1107/S2056989022007356/tx2055Isup4.cml

Structure factors: contains datablock(s) II. DOI: 10.1107/S2056989022007356/tx2055IIsup3.hkl

Supporting information file. DOI: 10.1107/S2056989022007356/tx2055IIsup5.cml

CCDC references: 2190978, 2190977

Additional supporting information:  crystallographic information; 3D view; checkCIF report

## Figures and Tables

**Table 1:** Hydrogen-bond geometry (Å, °) for (I)

*D*—H⋯*A*	*D*—H	H⋯*A*	*D*⋯*A*	*D*—H⋯*A*
N2—H2*A*⋯Br1^i^	0.82 (2)	2.75 (2)	3.507 (3)	154.4 (19)
N2—H2*A*⋯Br1*A* ^i^	0.82 (2)	2.73 (2)	3.493 (4)	155.1 (19)
N2—H2*B*⋯N3^ii^	0.84 (2)	2.24 (2)	3.0583 (18)	165 (2)
C5—H5*B*⋯N3^iii^	0.99	2.59	3.5426 (19)	160
C8—H8⋯O2^iv^	0.95	2.49	3.223 (2)	134
C12—H12⋯N3^v^	0.95	2.65	3.411 (2)	138
C16—H16⋯N3^vi^	0.95	2.62	3.5283 (19)	160
O2—H2*C*⋯O1	0.85 (1)	2.09 (2)	2.8739 (14)	153 (3)

Symmetry codes: (i) 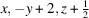 ; (ii) 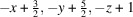 ; (iii) 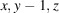 ; (iv) 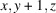 ; (v) 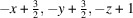 ; (vi) 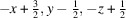 .

**Table 2:** Hydrogen-bond geometry (Å, °) for (II) *Cg*2 is the centroid of the C8–C13 phenyl ring.

*D*—H⋯*A*	*D*—H	H⋯*A*	*D*⋯*A*	*D*—H⋯*A*
N2—H2*A*⋯N4^i^	0.913 (13)	2.541 (13)	3.3713 (9)	151.6 (11)
N2—H2*B*⋯N4^ii^	0.916 (14)	2.495 (14)	3.2404 (10)	138.7 (11)
N2—H2*B*⋯O1^iii^	0.916 (14)	2.381 (13)	3.0650 (8)	131.5 (11)
N5—H5*A*⋯N3′^ii^	0.897 (14)	2.525 (14)	3.1431 (9)	126.6 (11)
N5—H5*B*⋯O1′^ii^	0.930 (14)	1.986 (14)	2.8853 (8)	162.3 (12)
N2′—H2*A*′⋯O1′^iv^	0.898 (13)	2.186 (13)	3.0608 (8)	164.4 (11)
N2′—H2*B*′⋯N2^iii^	0.902 (15)	2.681 (14)	3.1829 (8)	116.1 (10)
N2′—H2*B*′⋯O1	0.902 (15)	2.250 (15)	3.1373 (9)	167.5 (12)
N5′—H5*A*′⋯N3′^i^	0.887 (15)	2.102 (15)	2.9314 (8)	155.2 (13)
C9′—H9′⋯*Cg*2	0.95	2.93	3.7972 (5)	153

Symmetry codes: (i) 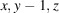 ; (ii) 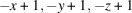 ; (iii) 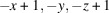 ; (iv) 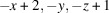 .

**Table 3:** Summary of short inter­atomic contacts (Å) in compound (I)

Contact	Distance	Symmetry operation
Br1*A*⋯H2*A*	2.73	*x*, 2 − *y*, − 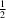 + *z*
O1⋯H2*C*	2.09	1 − *x*, *y*, 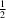 − *z*
O1⋯H19	2.40	*x*, −1 + *y*, *z*
N3⋯H2*B*	2.24	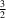 − *x*, 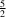 − *y*, 1 − *z*
H12⋯N3	2.65	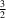 − *x*, 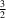 − *y*, 1 − *z*
N3⋯H16	2.62	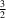 − *x*, 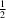 + *y*, 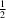 − *z*
H8⋯O2	2.49	*x*, 1 + *y*, *z*
H9⋯C18	2.69	1 − *x*, *y*, 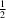 − *z*
O2⋯O1	2.87	1 − *x*, *y*, 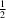 − *z*

**Table 4:** Summary of short inter­atomic contacts (Å) in compound (II)

Contact	Distance	Symmetry operation
O1⋯H2*B*′	2.25	*x*, *y*, *z*
H2*A*⋯N4	2.54	*x*, −1 + *y*, *z*
H2*B*⋯O1	2.38	1 − *x*, −*y*, 1 − *z*
H13⋯H5*A*	2.46	1 − *x*, 1 − *y*, 1 − *z*
N2⋯H2*B*′	2.68	1 − *x*, −*y*, 1 − *z*
H10⋯H12′	2.46	2 − *x*, 1 − *y*, −*z*
N4⋯H2*A*′	2.82	−1 + *x*, 1 + *y*, *z*
H5*B*⋯O1′	1.99	1 − *x*, 1 − *y*, 1 − *z*
H9⋯C12′	2.83	−1 + *x*, *y*, *z*
H12⋯N5′	2.89	*x*, 1 + *y*, *z*
H2*A*′⋯O1′	2.19	2 − *x*, −*y*, 1 − *z*
N3′⋯H5*A*′	2.10	1 + *x*, *y*, *z*
H10′⋯N4′	2.55	2 − *x*, 1 − *y*, −*z*
H12′⋯H12′	2.36	3 − *x*, 1 − *y*, −*z*

**Table 5:** Percentage contributions of inter­atomic contacts to the Hirshfeld surface for compound (I)

Contact	Percentage contribution
H⋯H	37.9
C⋯H/H⋯C	18.4
Br⋯H/H⋯Br	13.3
N⋯H/H⋯N	11.5
O⋯H/H⋯O	10.0
Br⋯C/C⋯Br	4.2
C⋯C	1.5
N⋯C/C⋯N	1.3
N⋯N	0.8
Br⋯Br	0.6
C⋯O/O⋯C	0.5

**Table 6:** Percentage contributions of inter­atomic contacts to the Hirshfeld surface for compound (II)

Contact	% contribution for IIA	% contribution for IIB
H⋯H	27.6	23.1
N⋯H/H⋯N	25.2	28.3
C⋯H/H⋯C	15.2	21.2
O⋯H/H⋯O	11.4	8.8
N⋯C/C⋯N	8.6	6.7
C⋯C	6.8	7.5
N⋯N	2.1	2.8
N⋯O/O⋯N	1.7	0.6
C⋯O/O⋯C	1.3	0.9

**Table 7:** Experimental details

	(I)	(II)
Crystal data		
Chemical formula	2C_18_H_14_BrN_3_O·H_2_O	C_13_H_9_N_5_O
*M*_r_	754.48	251.25
Crystal system, space group	Monoclinic, *C*2/*c*	Triclinic, *P* 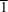
Temperature (K)	100	100
*a*, *b*, *c* (Å)	27.539 (3), 6.3430 (6), 21.3540 (19)	8.6444 (1), 8.9104 (2), 16.0902 (2)
α, β, γ (°)	90, 118.170 (12), 90	79.196 (1), 86.485 (1), 69.003 (2)
*V* (Å^3^)	3288.2 (6)	1136.52 (4)
*Z*	4	4
Radiation type	Synchrotron, λ = 0.74500 Å	Mo *K*α
μ (mm^−1^)	2.83	0.10
Crystal size (mm)	0.10 × 0.07 × 0.05	0.15 × 0.12 × 0.06

Data collection		
Diffractometer	Rayonix SX-165 CCD	XtaLAB Synergy, Dualflex, HyPix
Absorption correction	Multi-scan (*SCALA*; Evans, 2006 ▸)	Multi-scan (*CrysAlis PRO*; Rigaku OD, 2021 ▸)
*T*_min_, *T*_max_	0.742, 0.851	0.972, 0.980
No. of measured, independent and observed [*I* > 2σ(*I*)] reflections	28485, 4492, 4047	88754, 9666, 8561
*R*_int_	0.025	0.029
(sin θ/λ)_max_ (Å^−1^)	0.692	0.816

Refinement		
*R*[*F* ^2^ > 2σ(*F* ^2^)], *wR*(*F* ^2^), *S*	0.031, 0.086, 1.06	0.039, 0.118, 1.03
No. of reflections	4492	9666
No. of parameters	233	363
No. of restraints	2	0
H-atom treatment	H atoms treated by a mixture of independent and constrained refinement	H atoms treated by a mixture of independent and constrained refinement
Δρ_max_, Δρ_min_ (e Å^−3^)	0.45, −0.43	0.54, −0.31

Computer programs: *Marccd* (Doyle, 2011 ▸), *CrysAlis PRO* (Rigaku OD, 2021 ▸), *iMosflm* (Battye *et al.*, 2011 ▸), *SHELXT* (Sheldrick, 2015*a* ▸), *SHELXL* (Sheldrick, 2015*b* ▸), *ORTEP-3 for Windows* (Farrugia, 2012 ▸), and *PLATON* (Spek, 2020 ▸).

## supplementary crystallographic information

### 2-Amino-4-(4-bromophenyl)-6-oxo-1-phenyl-1,4,5,6-tetrahydropyridine-3-carbonitrile hemihydrate (I) Crystal data

**Table:**

2C_18_H_14_BrN_3_O·H_2_O	*F*(000) = 1528
*M**_r_* = 754.48	*D*_x_ = 1.524 Mg m^−^^3^
Monoclinic, *C*2/*c*	Synchrotron radiation, λ = 0.74500 Å
*a* = 27.539 (3) Å	Cell parameters from 1000 reflections
*b* = 6.3430 (6) Å	θ = 1.8–24.0°
*c* = 21.3540 (19) Å	µ = 2.83 mm^−^^1^
β = 118.170 (12)°	*T* = 100 K
*V* = 3288.2 (6) Å^3^	Prism, yellow
*Z* = 4	0.10 × 0.07 × 0.05 mm

### 2-Amino-4-(4-bromophenyl)-6-oxo-1-phenyl-1,4,5,6-tetrahydropyridine-3-carbonitrile hemihydrate (I) Data collection

**Table:**

Rayonix SX-165 CCD diffractometer	4047 reflections with *I* > 2σ(*I*)
/f scan	*R*_int_ = 0.025
Absorption correction: multi-scan (*SCALA*; Evans, 2006)	θ_max_ = 31.0°, θ_min_ = 1.8°
*T*_min_ = 0.742, *T*_max_ = 0.851	*h* = −38→38
28485 measured reflections	*k* = −8→8
4492 independent reflections	*l* = −29→29

### 2-Amino-4-(4-bromophenyl)-6-oxo-1-phenyl-1,4,5,6-tetrahydropyridine-3-carbonitrile hemihydrate (I) Refinement

**Table:**

Refinement on *F*^2^	Hydrogen site location: mixed
Least-squares matrix: full	H atoms treated by a mixture of independent and constrained refinement
*R*[*F*^2^ > 2σ(*F*^2^)] = 0.031	*w* = 1/[σ^2^(*F*_o_^2^) + (0.0485*P*)^2^ + 2.5862*P*] where *P* = (*F*_o_^2^ + 2*F*_c_^2^)/3
*wR*(*F*^2^) = 0.086	(Δ/σ)_max_ = 0.001
*S* = 1.06	Δρ_max_ = 0.45 e Å^−^^3^
4492 reflections	Δρ_min_ = −0.43 e Å^−^^3^
233 parameters	Extinction correction: SHELXL-2018/3 (Sheldrick, 2015b), Fc^*^=kFc[1+0.001xFc^2^λ^3^/sin(2θ)]^-1/4^
2 restraints	Extinction coefficient: 0.0066 (5)

### 2-Amino-4-(4-bromophenyl)-6-oxo-1-phenyl-1,4,5,6-tetrahydropyridine-3-carbonitrile hemihydrate (I) Special details

**Table:**

Geometry. All esds (except the esd in the dihedral angle between two l.s. planes) are estimated using the full covariance matrix. The cell esds are taken into account individually in the estimation of esds in distances, angles and torsion angles; correlations between esds in cell parameters are only used when they are defined by crystal symmetry. An approximate (isotropic) treatment of cell esds is used for estimating esds involving l.s. planes.

### 2-Amino-4-(4-bromophenyl)-6-oxo-1-phenyl-1,4,5,6-tetrahydropyridine-3-carbonitrile hemihydrate (I) Fractional atomic coordinates and isotropic or equivalent isotropic displacement parameters (Å^2^)

**Table:**

	*x*	*y*	*z*	*U*_iso_*/*U*_eq_	Occ. (<1)
Br1	0.58033 (10)	1.0754 (4)	0.04138 (12)	0.0362 (3)	0.59 (2)
Br1A	0.5838 (2)	1.1121 (17)	0.0450 (2)	0.0521 (7)	0.41 (2)
O1	0.61090 (5)	0.37633 (17)	0.30715 (7)	0.0337 (3)	
N1	0.63725 (5)	0.65818 (18)	0.38065 (6)	0.0224 (2)	
N2	0.66784 (6)	0.9297 (2)	0.46401 (7)	0.0259 (2)	
H2A	0.6410 (10)	0.904 (3)	0.4699 (12)	0.031*	
H2B	0.6843 (9)	1.044 (3)	0.4797 (11)	0.031*	
N3	0.78997 (5)	1.13330 (19)	0.47344 (7)	0.0273 (2)	
C2	0.67632 (6)	0.8155 (2)	0.41704 (7)	0.0221 (2)	
C3	0.71957 (6)	0.8467 (2)	0.40309 (7)	0.0228 (2)	
C4	0.72370 (6)	0.7256 (2)	0.34490 (7)	0.0222 (2)	
H4	0.763385	0.716345	0.356821	0.027*	
C5	0.70242 (6)	0.5023 (2)	0.34527 (8)	0.0248 (3)	
H5A	0.700533	0.422449	0.304338	0.030*	
H5B	0.728708	0.428933	0.389238	0.030*	
C6	0.64645 (6)	0.5040 (2)	0.34127 (8)	0.0248 (3)	
C7	0.58549 (6)	0.6519 (2)	0.38258 (7)	0.0249 (3)	
C8	0.54681 (7)	0.8076 (3)	0.34787 (9)	0.0339 (3)	
H8	0.554201	0.918309	0.323626	0.041*	
C9	0.49690 (8)	0.7984 (3)	0.34924 (11)	0.0446 (4)	
H9	0.470156	0.905529	0.326486	0.053*	
C10	0.48580 (7)	0.6351 (3)	0.38334 (11)	0.0442 (4)	
H10	0.451308	0.629490	0.383299	0.053*	
C11	0.52464 (7)	0.4800 (3)	0.41753 (10)	0.0382 (4)	
H11	0.516806	0.367789	0.440849	0.046*	
C12	0.57518 (7)	0.4881 (2)	0.41780 (9)	0.0304 (3)	
H12	0.602285	0.382964	0.441729	0.037*	
C13	0.75857 (6)	1.0032 (2)	0.44177 (7)	0.0230 (2)	
C14	0.69159 (6)	0.8247 (2)	0.27130 (7)	0.0222 (2)	
C15	0.69605 (6)	0.7419 (2)	0.21353 (8)	0.0284 (3)	
H15	0.721123	0.629945	0.220994	0.034*	
C16	0.66441 (7)	0.8207 (3)	0.14545 (8)	0.0320 (3)	
H16	0.667074	0.761790	0.106281	0.038*	
C17	0.62892 (6)	0.9867 (3)	0.13569 (8)	0.0293 (3)	
C18	0.62500 (6)	1.0777 (2)	0.19181 (8)	0.0264 (3)	
H18	0.601436	1.194973	0.184383	0.032*	
C19	0.65621 (6)	0.9943 (2)	0.25941 (7)	0.0235 (3)	
H19	0.653345	1.054266	0.298300	0.028*	
O2	0.500000	0.2217 (3)	0.250000	0.0497 (5)	
H2C	0.5274 (8)	0.305 (4)	0.2670 (16)	0.075*	

### 2-Amino-4-(4-bromophenyl)-6-oxo-1-phenyl-1,4,5,6-tetrahydropyridine-3-carbonitrile hemihydrate (I) Atomic displacement parameters (Å^2^)

**Table:**

	*U*^11^	*U*^22^	*U*^33^	*U*^12^	*U*^13^	*U*^23^
Br1	0.0326 (4)	0.0536 (6)	0.0240 (3)	0.0061 (6)	0.0149 (3)	0.0099 (3)
Br1A	0.0486 (12)	0.0873 (19)	0.0298 (7)	0.0336 (9)	0.0262 (7)	0.0238 (8)
O1	0.0301 (6)	0.0284 (5)	0.0446 (6)	−0.0049 (4)	0.0193 (5)	−0.0142 (4)
N1	0.0219 (5)	0.0217 (5)	0.0239 (5)	−0.0008 (4)	0.0109 (4)	−0.0025 (4)
N2	0.0274 (6)	0.0263 (5)	0.0266 (6)	−0.0044 (5)	0.0149 (5)	−0.0058 (4)
N3	0.0290 (6)	0.0255 (5)	0.0276 (6)	−0.0020 (5)	0.0133 (5)	−0.0005 (4)
C2	0.0238 (6)	0.0203 (5)	0.0205 (5)	0.0009 (4)	0.0090 (5)	0.0005 (4)
C3	0.0235 (6)	0.0221 (5)	0.0216 (6)	−0.0002 (5)	0.0096 (5)	−0.0003 (4)
C4	0.0220 (6)	0.0212 (5)	0.0239 (6)	0.0014 (4)	0.0112 (5)	−0.0004 (4)
C5	0.0259 (7)	0.0200 (5)	0.0298 (6)	0.0031 (5)	0.0142 (5)	0.0004 (5)
C6	0.0259 (7)	0.0204 (5)	0.0278 (6)	0.0009 (5)	0.0126 (5)	−0.0015 (5)
C7	0.0208 (6)	0.0277 (6)	0.0254 (6)	−0.0014 (5)	0.0102 (5)	−0.0055 (5)
C8	0.0285 (8)	0.0374 (8)	0.0329 (7)	0.0058 (6)	0.0122 (6)	−0.0010 (6)
C9	0.0278 (8)	0.0549 (10)	0.0469 (10)	0.0111 (7)	0.0143 (7)	−0.0055 (8)
C10	0.0258 (8)	0.0594 (11)	0.0507 (10)	−0.0063 (7)	0.0207 (8)	−0.0189 (9)
C11	0.0336 (8)	0.0426 (9)	0.0448 (9)	−0.0120 (7)	0.0238 (7)	−0.0119 (7)
C12	0.0277 (7)	0.0303 (7)	0.0350 (7)	−0.0053 (6)	0.0162 (6)	−0.0049 (6)
C13	0.0247 (6)	0.0229 (6)	0.0221 (6)	0.0020 (5)	0.0116 (5)	0.0021 (5)
C14	0.0230 (6)	0.0213 (5)	0.0241 (6)	−0.0007 (5)	0.0125 (5)	−0.0010 (4)
C15	0.0299 (7)	0.0300 (6)	0.0290 (7)	0.0052 (5)	0.0169 (6)	−0.0007 (5)
C16	0.0325 (8)	0.0429 (8)	0.0259 (7)	0.0041 (6)	0.0182 (6)	−0.0009 (6)
C17	0.0271 (7)	0.0396 (7)	0.0227 (6)	0.0028 (6)	0.0132 (5)	0.0068 (6)
C18	0.0275 (7)	0.0263 (6)	0.0268 (6)	0.0026 (5)	0.0139 (6)	0.0036 (5)
C19	0.0274 (7)	0.0218 (6)	0.0232 (6)	0.0002 (5)	0.0135 (5)	−0.0009 (5)
O2	0.0349 (10)	0.0329 (9)	0.0631 (13)	0.000	0.0082 (9)	0.000

### 2-Amino-4-(4-bromophenyl)-6-oxo-1-phenyl-1,4,5,6-tetrahydropyridine-3-carbonitrile hemihydrate (I) Geometric parameters (Å, º)

**Table:**

Br1—C17	1.902 (3)	C8—C9	1.390 (2)
Br1A—C17	1.912 (4)	C8—H8	0.9500
O1—C6	1.2131 (18)	C9—C10	1.381 (3)
N1—C6	1.3886 (17)	C9—H9	0.9500
N1—C2	1.4027 (17)	C10—C11	1.381 (3)
N1—C7	1.4460 (18)	C10—H10	0.9500
N2—C2	1.3440 (17)	C11—C12	1.390 (2)
N2—H2A	0.82 (2)	C11—H11	0.9500
N2—H2B	0.84 (2)	C12—H12	0.9500
N3—C13	1.1551 (19)	C14—C19	1.3921 (18)
C2—C3	1.3716 (19)	C14—C15	1.3986 (18)
C3—C13	1.4080 (19)	C15—C16	1.388 (2)
C3—C4	1.5101 (18)	C15—H15	0.9500
C4—C14	1.5278 (19)	C16—C17	1.384 (2)
C4—C5	1.5347 (18)	C16—H16	0.9500
C4—H4	1.0000	C17—C18	1.380 (2)
C5—C6	1.5031 (19)	C18—C19	1.390 (2)
C5—H5A	0.9900	C18—H18	0.9500
C5—H5B	0.9900	C19—H19	0.9500
C7—C8	1.383 (2)	O2—H2C	0.849 (10)
C7—C12	1.389 (2)	O2—H2C^i^	0.849 (10)
			
C6—N1—C2	121.67 (12)	C10—C9—H9	119.7
C6—N1—C7	117.52 (11)	C8—C9—H9	119.7
C2—N1—C7	120.82 (11)	C9—C10—C11	120.26 (16)
C2—N2—H2A	119.6 (14)	C9—C10—H10	119.9
C2—N2—H2B	120.8 (15)	C11—C10—H10	119.9
H2A—N2—H2B	118 (2)	C10—C11—C12	120.00 (17)
N2—C2—C3	123.97 (13)	C10—C11—H11	120.0
N2—C2—N1	116.02 (12)	C12—C11—H11	120.0
C3—C2—N1	120.00 (12)	C7—C12—C11	119.05 (16)
C2—C3—C13	118.33 (12)	C7—C12—H12	120.5
C2—C3—C4	121.03 (12)	C11—C12—H12	120.5
C13—C3—C4	120.55 (12)	N3—C13—C3	179.02 (15)
C3—C4—C14	113.92 (11)	C19—C14—C15	118.33 (13)
C3—C4—C5	106.75 (11)	C19—C14—C4	121.39 (11)
C14—C4—C5	110.35 (11)	C15—C14—C4	120.26 (12)
C3—C4—H4	108.6	C16—C15—C14	121.04 (13)
C14—C4—H4	108.6	C16—C15—H15	119.5
C5—C4—H4	108.6	C14—C15—H15	119.5
C6—C5—C4	112.13 (11)	C17—C16—C15	118.77 (13)
C6—C5—H5A	109.2	C17—C16—H16	120.6
C4—C5—H5A	109.2	C15—C16—H16	120.6
C6—C5—H5B	109.2	C18—C17—C16	121.80 (13)
C4—C5—H5B	109.2	C18—C17—Br1	119.43 (14)
H5A—C5—H5B	107.9	C16—C17—Br1	118.58 (14)
O1—C6—N1	120.51 (13)	C18—C17—Br1A	115.2 (2)
O1—C6—C5	122.98 (12)	C16—C17—Br1A	123.0 (2)
N1—C6—C5	116.49 (12)	C17—C18—C19	118.64 (13)
C8—C7—C12	121.46 (14)	C17—C18—H18	120.7
C8—C7—N1	119.04 (13)	C19—C18—H18	120.7
C12—C7—N1	119.49 (13)	C18—C19—C14	121.34 (12)
C7—C8—C9	118.56 (17)	C18—C19—H19	119.3
C7—C8—H8	120.7	C14—C19—H19	119.3
C9—C8—H8	120.7	H2C—O2—H2C^i^	103 (4)
C10—C9—C8	120.66 (17)		
			
C6—N1—C2—N2	−167.48 (13)	C12—C7—C8—C9	0.5 (2)
C7—N1—C2—N2	12.90 (18)	N1—C7—C8—C9	179.12 (14)
C6—N1—C2—C3	13.26 (19)	C7—C8—C9—C10	−1.3 (3)
C7—N1—C2—C3	−166.35 (13)	C8—C9—C10—C11	0.9 (3)
N2—C2—C3—C13	2.1 (2)	C9—C10—C11—C12	0.1 (3)
N1—C2—C3—C13	−178.68 (12)	C8—C7—C12—C11	0.5 (2)
N2—C2—C3—C4	−174.42 (13)	N1—C7—C12—C11	−178.07 (13)
N1—C2—C3—C4	4.77 (19)	C10—C11—C12—C7	−0.8 (2)
C2—C3—C4—C14	85.13 (15)	C3—C4—C14—C19	−8.24 (18)
C13—C3—C4—C14	−91.35 (15)	C5—C4—C14—C19	111.81 (14)
C2—C3—C4—C5	−36.93 (16)	C3—C4—C14—C15	173.58 (13)
C13—C3—C4—C5	146.59 (12)	C5—C4—C14—C15	−66.37 (16)
C3—C4—C5—C6	52.57 (15)	C19—C14—C15—C16	−2.6 (2)
C14—C4—C5—C6	−71.70 (14)	C4—C14—C15—C16	175.64 (14)
C2—N1—C6—O1	−176.04 (13)	C14—C15—C16—C17	1.3 (2)
C7—N1—C6—O1	3.6 (2)	C15—C16—C17—C18	1.2 (2)
C2—N1—C6—C5	5.58 (18)	C15—C16—C17—Br1	−173.74 (14)
C7—N1—C6—C5	−174.79 (12)	C15—C16—C17—Br1A	179.2 (4)
C4—C5—C6—O1	141.73 (14)	C16—C17—C18—C19	−2.4 (2)
C4—C5—C6—N1	−39.93 (17)	Br1—C17—C18—C19	172.52 (13)
C6—N1—C7—C8	−110.24 (15)	Br1A—C17—C18—C19	179.5 (3)
C2—N1—C7—C8	69.39 (18)	C17—C18—C19—C14	1.1 (2)
C6—N1—C7—C12	68.39 (17)	C15—C14—C19—C18	1.4 (2)
C2—N1—C7—C12	−111.98 (15)	C4—C14—C19—C18	−176.83 (13)

Symmetry code: (i) −*x*+1, *y*, −*z*+1/2.

### 2-Amino-4-(4-bromophenyl)-6-oxo-1-phenyl-1,4,5,6-tetrahydropyridine-3-carbonitrile hemihydrate (I) Hydrogen-bond geometry (Å, º)

**Table:**

*D*—H···*A*	*D*—H	H···*A*	*D*···*A*	*D*—H···*A*
N2—H2*A*···Br1^ii^	0.82 (2)	2.75 (2)	3.507 (3)	154.4 (19)
N2—H2*A*···Br1*A*^ii^	0.82 (2)	2.73 (2)	3.493 (4)	155.1 (19)
N2—H2*B*···N3^iii^	0.84 (2)	2.24 (2)	3.0583 (18)	165 (2)
C5—H5*B*···N3^iv^	0.99	2.59	3.5426 (19)	160
C8—H8···O2^v^	0.95	2.49	3.223 (2)	134
C12—H12···N3^vi^	0.95	2.65	3.411 (2)	138
C16—H16···N3^vii^	0.95	2.62	3.5283 (19)	160
O2—H2*C*···O1	0.85 (1)	2.09 (2)	2.8739 (14)	153 (3)

Symmetry codes: (ii) *x*, −*y*+2, *z*+1/2; (iii) −*x*+3/2, −*y*+5/2, −*z*+1; (iv) *x*, *y*−1, *z*; (v) *x*, *y*+1, *z*; (vi) −*x*+3/2, −*y*+3/2, −*z*+1; (vii) −*x*+3/2, *y*−1/2, −*z*+1/2.

### 1,6-Diamino-2-oxo-4-phenyl-1,2-dihydropyridine-3,5-dicarbonitrile (II) Crystal data

**Table:**

C_13_H_9_N_5_O	*Z* = 4
*M**_r_* = 251.25	*F*(000) = 520
Triclinic, *P*1	*D*_x_ = 1.468 Mg m^−^^3^
*a* = 8.6444 (1) Å	Mo *K*α radiation, λ = 0.71073 Å
*b* = 8.9104 (2) Å	Cell parameters from 60859 reflections
*c* = 16.0902 (2) Å	θ = 2.5–35.6°
α = 79.196 (1)°	µ = 0.10 mm^−^^1^
β = 86.485 (1)°	*T* = 100 K
γ = 69.003 (2)°	Prism, colourless
*V* = 1136.52 (4) Å^3^	0.15 × 0.12 × 0.06 mm

### 1,6-Diamino-2-oxo-4-phenyl-1,2-dihydropyridine-3,5-dicarbonitrile (II) Data collection

**Table:**

XtaLAB Synergy, Dualflex, HyPix diffractometer	8561 reflections with *I* > 2σ(*I*)
Radiation source: micro-focus sealed X-ray tube	*R*_int_ = 0.029
φ and ω scans	θ_max_ = 35.4°, θ_min_ = 2.5°
Absorption correction: multi-scan (CrysAlisPro; Rigaku OD, 2021)	*h* = −14→13
*T*_min_ = 0.972, *T*_max_ = 0.980	*k* = −14→14
88754 measured reflections	*l* = −25→26
9666 independent reflections	

### 1,6-Diamino-2-oxo-4-phenyl-1,2-dihydropyridine-3,5-dicarbonitrile (II) Refinement

**Table:**

Refinement on *F*^2^	0 restraints
Least-squares matrix: full	Hydrogen site location: mixed
*R*[*F*^2^ > 2σ(*F*^2^)] = 0.039	H atoms treated by a mixture of independent and constrained refinement
*wR*(*F*^2^) = 0.118	*w* = 1/[σ^2^(*F*_o_^2^) + (0.0719*P*)^2^ + 0.2449*P*] where *P* = (*F*_o_^2^ + 2*F*_c_^2^)/3
*S* = 1.03	(Δ/σ)_max_ = 0.001
9666 reflections	Δρ_max_ = 0.54 e Å^−^^3^
363 parameters	Δρ_min_ = −0.31 e Å^−^^3^

### 1,6-Diamino-2-oxo-4-phenyl-1,2-dihydropyridine-3,5-dicarbonitrile (II) Special details

**Table:**

Geometry. All esds (except the esd in the dihedral angle between two l.s. planes) are estimated using the full covariance matrix. The cell esds are taken into account individually in the estimation of esds in distances, angles and torsion angles; correlations between esds in cell parameters are only used when they are defined by crystal symmetry. An approximate (isotropic) treatment of cell esds is used for estimating esds involving l.s. planes.

### 1,6-Diamino-2-oxo-4-phenyl-1,2-dihydropyridine-3,5-dicarbonitrile (II) Fractional atomic coordinates and isotropic or equivalent isotropic displacement parameters (Å^2^)

**Table:**

	*x*	*y*	*z*	*U*_iso_*/*U*_eq_	
C2	0.49789 (8)	0.24937 (8)	0.36884 (4)	0.01381 (11)	
C3	0.53566 (8)	0.37133 (8)	0.30738 (4)	0.01370 (11)	
C4	0.48775 (8)	0.53309 (8)	0.31837 (4)	0.01268 (10)	
C5	0.39202 (8)	0.57960 (7)	0.38978 (4)	0.01268 (10)	
C6	0.33478 (8)	0.46771 (8)	0.44558 (4)	0.01249 (10)	
C7	0.63071 (9)	0.31152 (8)	0.23688 (4)	0.01525 (11)	
C8	0.53483 (8)	0.65513 (8)	0.25605 (4)	0.01337 (11)	
C9	0.50427 (10)	0.67233 (8)	0.16981 (4)	0.01755 (12)	
H9	0.453811	0.605695	0.151080	0.021*	
C10	0.54766 (11)	0.78697 (9)	0.11127 (5)	0.02109 (14)	
H10	0.526489	0.798239	0.052760	0.025*	
C11	0.62188 (10)	0.88513 (9)	0.13807 (5)	0.02022 (13)	
H11	0.652279	0.962460	0.097961	0.024*	
C12	0.65120 (9)	0.86934 (8)	0.22373 (5)	0.01812 (12)	
H12	0.700559	0.937029	0.242277	0.022*	
C13	0.60864 (9)	0.75488 (8)	0.28249 (4)	0.01520 (11)	
H13	0.629797	0.744330	0.340946	0.018*	
C14	0.34605 (8)	0.74045 (8)	0.40775 (4)	0.01414 (11)	
N1	0.38849 (7)	0.30955 (7)	0.43300 (4)	0.01336 (10)	
N2	0.33246 (8)	0.19935 (7)	0.49062 (4)	0.01632 (11)	
H2A	0.3085 (16)	0.1388 (16)	0.4569 (8)	0.027 (3)*	
H2B	0.4202 (17)	0.1317 (16)	0.5247 (8)	0.029 (3)*	
N3	0.70345 (9)	0.25693 (8)	0.18045 (4)	0.02017 (12)	
N4	0.31176 (8)	0.86837 (7)	0.42590 (4)	0.01799 (11)	
N5	0.23470 (8)	0.50589 (7)	0.51032 (4)	0.01504 (10)	
H5A	0.1960 (17)	0.4294 (17)	0.5378 (8)	0.030 (3)*	
H5B	0.1916 (17)	0.6134 (17)	0.5190 (9)	0.031 (3)*	
O1	0.55120 (7)	0.10126 (6)	0.36855 (3)	0.01817 (10)	
C2'	0.97520 (8)	0.16536 (7)	0.35832 (4)	0.01262 (11)	
C3'	1.02820 (8)	0.27412 (7)	0.29632 (4)	0.01245 (10)	
C4'	1.08425 (8)	0.23854 (7)	0.21668 (4)	0.01188 (10)	
C5'	1.09636 (8)	0.08798 (7)	0.19804 (4)	0.01221 (10)	
C6'	1.04791 (8)	−0.02418 (7)	0.25855 (4)	0.01190 (10)	
C7'	1.02248 (9)	0.42022 (8)	0.32183 (4)	0.01393 (11)	
C8'	1.12573 (5)	0.36189 (4)	0.15294 (3)	0.01286 (11)	
C9'	1.00656 (4)	0.51639 (5)	0.13008 (3)	0.01666 (12)	
H9'	0.899783	0.541883	0.154979	0.020*	
C10'	1.04361 (6)	0.63360 (4)	0.07079 (3)	0.02107 (14)	
H10'	0.962158	0.739194	0.055167	0.025*	
C11'	1.19983 (6)	0.59630 (5)	0.03436 (3)	0.02295 (15)	
H11'	1.225153	0.676408	−0.006158	0.028*	
C12'	1.31900 (5)	0.44180 (6)	0.05723 (3)	0.02250 (15)	
H12'	1.425776	0.416311	0.032329	0.027*	
C13'	1.28195 (5)	0.32459 (4)	0.11652 (3)	0.01777 (12)	
H13'	1.363404	0.218998	0.132141	0.021*	
C14'	1.14171 (9)	0.04541 (8)	0.11662 (4)	0.01460 (11)	
N1'	0.98460 (7)	0.01996 (6)	0.33357 (3)	0.01239 (10)	
N2'	0.93299 (8)	−0.09390 (7)	0.39068 (4)	0.01582 (11)	
H2A'	0.9758 (16)	−0.1024 (15)	0.4416 (8)	0.022 (3)*	
H2B'	0.8215 (18)	−0.0479 (17)	0.3919 (9)	0.032 (3)*	
N3'	1.01668 (9)	0.53667 (7)	0.34567 (4)	0.01855 (12)	
N4'	1.17585 (9)	0.00229 (8)	0.05229 (4)	0.02098 (12)	
N5'	1.05814 (8)	−0.16991 (7)	0.24521 (4)	0.01556 (11)	
H5A'	1.0282 (19)	−0.2369 (18)	0.2854 (9)	0.039 (4)*	
H5B'	1.0896 (15)	−0.2001 (15)	0.1954 (8)	0.023 (3)*	
O1'	0.92331 (7)	0.18943 (6)	0.42983 (3)	0.01749 (10)	

### 1,6-Diamino-2-oxo-4-phenyl-1,2-dihydropyridine-3,5-dicarbonitrile (II) Atomic displacement parameters (Å^2^)

**Table:**

	*U*^11^	*U*^22^	*U*^33^	*U*^12^	*U*^13^	*U*^23^
C2	0.0160 (3)	0.0122 (2)	0.0135 (2)	−0.0053 (2)	0.0029 (2)	−0.00348 (19)
C3	0.0169 (3)	0.0119 (2)	0.0126 (2)	−0.0055 (2)	0.0032 (2)	−0.00320 (19)
C4	0.0144 (3)	0.0120 (2)	0.0119 (2)	−0.0050 (2)	0.00073 (19)	−0.00226 (18)
C5	0.0156 (3)	0.0106 (2)	0.0122 (2)	−0.0051 (2)	0.00160 (19)	−0.00266 (18)
C6	0.0145 (3)	0.0116 (2)	0.0115 (2)	−0.0046 (2)	0.00057 (19)	−0.00262 (18)
C7	0.0173 (3)	0.0134 (2)	0.0150 (3)	−0.0056 (2)	0.0018 (2)	−0.0027 (2)
C8	0.0160 (3)	0.0117 (2)	0.0121 (2)	−0.0049 (2)	0.00167 (19)	−0.00189 (18)
C9	0.0234 (3)	0.0156 (3)	0.0129 (3)	−0.0059 (2)	−0.0002 (2)	−0.0026 (2)
C10	0.0292 (4)	0.0173 (3)	0.0127 (3)	−0.0047 (3)	0.0022 (2)	−0.0006 (2)
C11	0.0244 (3)	0.0141 (3)	0.0183 (3)	−0.0046 (2)	0.0068 (2)	0.0002 (2)
C12	0.0199 (3)	0.0147 (3)	0.0204 (3)	−0.0078 (2)	0.0051 (2)	−0.0028 (2)
C13	0.0177 (3)	0.0144 (3)	0.0146 (3)	−0.0073 (2)	0.0020 (2)	−0.0025 (2)
C14	0.0156 (3)	0.0135 (2)	0.0138 (2)	−0.0059 (2)	0.0018 (2)	−0.00275 (19)
N1	0.0169 (2)	0.0103 (2)	0.0131 (2)	−0.00544 (18)	0.00382 (18)	−0.00272 (17)
N2	0.0212 (3)	0.0126 (2)	0.0161 (2)	−0.0083 (2)	0.0051 (2)	−0.00171 (18)
N3	0.0222 (3)	0.0194 (3)	0.0181 (3)	−0.0063 (2)	0.0046 (2)	−0.0052 (2)
N4	0.0207 (3)	0.0149 (2)	0.0200 (3)	−0.0077 (2)	0.0039 (2)	−0.0052 (2)
N5	0.0189 (3)	0.0128 (2)	0.0133 (2)	−0.00552 (19)	0.00438 (19)	−0.00362 (17)
O1	0.0235 (2)	0.0112 (2)	0.0197 (2)	−0.00596 (18)	0.00526 (19)	−0.00440 (16)
C2'	0.0176 (3)	0.0089 (2)	0.0115 (2)	−0.0048 (2)	0.00219 (19)	−0.00237 (18)
C3'	0.0179 (3)	0.0091 (2)	0.0115 (2)	−0.0061 (2)	0.00252 (19)	−0.00242 (18)
C4'	0.0144 (3)	0.0097 (2)	0.0116 (2)	−0.00459 (19)	0.00144 (19)	−0.00171 (18)
C5'	0.0168 (3)	0.0100 (2)	0.0101 (2)	−0.0053 (2)	0.00269 (19)	−0.00226 (18)
C6'	0.0149 (3)	0.0095 (2)	0.0115 (2)	−0.00460 (19)	0.00159 (19)	−0.00249 (18)
C7'	0.0186 (3)	0.0113 (2)	0.0123 (2)	−0.0063 (2)	0.0015 (2)	−0.00144 (19)
C8'	0.0178 (3)	0.0108 (2)	0.0112 (2)	−0.0070 (2)	0.00243 (19)	−0.00177 (18)
C9'	0.0212 (3)	0.0126 (2)	0.0142 (3)	−0.0052 (2)	0.0010 (2)	0.0004 (2)
C10'	0.0331 (4)	0.0148 (3)	0.0148 (3)	−0.0099 (3)	−0.0004 (3)	0.0017 (2)
C11'	0.0400 (4)	0.0200 (3)	0.0153 (3)	−0.0195 (3)	0.0056 (3)	−0.0022 (2)
C12'	0.0300 (4)	0.0217 (3)	0.0226 (3)	−0.0175 (3)	0.0122 (3)	−0.0076 (3)
C13'	0.0199 (3)	0.0152 (3)	0.0208 (3)	−0.0094 (2)	0.0068 (2)	−0.0051 (2)
C14'	0.0190 (3)	0.0113 (2)	0.0133 (2)	−0.0057 (2)	0.0020 (2)	−0.00160 (19)
N1'	0.0184 (2)	0.0086 (2)	0.0109 (2)	−0.00621 (18)	0.00355 (17)	−0.00196 (16)
N2'	0.0235 (3)	0.0116 (2)	0.0138 (2)	−0.0093 (2)	0.0063 (2)	−0.00122 (17)
N3'	0.0277 (3)	0.0133 (2)	0.0168 (2)	−0.0098 (2)	0.0017 (2)	−0.00329 (19)
N4'	0.0300 (3)	0.0178 (3)	0.0148 (2)	−0.0079 (2)	0.0039 (2)	−0.0045 (2)
N5'	0.0239 (3)	0.0105 (2)	0.0146 (2)	−0.0085 (2)	0.0047 (2)	−0.00454 (18)
O1'	0.0286 (3)	0.0125 (2)	0.0116 (2)	−0.00763 (18)	0.00628 (18)	−0.00403 (15)

### 1,6-Diamino-2-oxo-4-phenyl-1,2-dihydropyridine-3,5-dicarbonitrile (II) Geometric parameters (Å, º)

**Table:**

C2—O1	1.2330 (8)	C2'—O1'	1.2376 (7)
C2—N1	1.4044 (8)	C2'—N1'	1.3992 (8)
C2—C3	1.4412 (9)	C2'—C3'	1.4296 (8)
C3—C4	1.3925 (9)	C3'—C4'	1.3939 (9)
C3—C7	1.4300 (9)	C3'—C7'	1.4204 (9)
C4—C5	1.4117 (9)	C4'—C5'	1.3954 (8)
C4—C8	1.4872 (9)	C4'—C8'	1.4843
C5—C6	1.4172 (9)	C5'—C6'	1.4167 (8)
C5—C14	1.4243 (9)	C5'—C14'	1.4251 (9)
C6—N5	1.3268 (8)	C6'—N5'	1.3266 (8)
C6—N1	1.3672 (8)	C6'—N1'	1.3617 (8)
C7—N3	1.1545 (9)	C7'—N3'	1.1558 (8)
C8—C9	1.3982 (9)	C8'—C9'	1.3900
C8—C13	1.3999 (9)	C8'—C13'	1.3900
C9—C10	1.3935 (10)	C9'—C10'	1.3900
C9—H9	0.9500	C9'—H9'	0.9500
C10—C11	1.3928 (12)	C10'—C11'	1.3900
C10—H10	0.9500	C10'—H10'	0.9500
C11—C12	1.3887 (11)	C11'—C12'	1.3900
C11—H11	0.9500	C11'—H11'	0.9500
C12—C13	1.3907 (9)	C12'—C13'	1.3900
C12—H12	0.9500	C12'—H12'	0.9500
C13—H13	0.9500	C13'—H13'	0.9500
C14—N4	1.1595 (9)	C14'—N4'	1.1561 (9)
N1—N2	1.4151 (8)	N1'—N2'	1.4132 (7)
N2—H2A	0.913 (13)	N2'—H2A'	0.898 (13)
N2—H2B	0.916 (14)	N2'—H2B'	0.902 (15)
N5—H5A	0.897 (14)	N5'—H5A'	0.887 (15)
N5—H5B	0.930 (14)	N5'—H5B'	0.889 (12)
			
O1—C2—N1	118.98 (6)	O1'—C2'—N1'	118.89 (6)
O1—C2—C3	125.76 (6)	O1'—C2'—C3'	125.90 (6)
N1—C2—C3	115.25 (5)	N1'—C2'—C3'	115.20 (5)
C4—C3—C7	123.26 (6)	C4'—C3'—C7'	122.09 (6)
C4—C3—C2	121.92 (6)	C4'—C3'—C2'	122.48 (5)
C7—C3—C2	114.77 (5)	C7'—C3'—C2'	115.42 (5)
C3—C4—C5	118.58 (6)	C3'—C4'—C5'	118.84 (5)
C3—C4—C8	120.96 (6)	C3'—C4'—C8'	119.79 (5)
C5—C4—C8	120.46 (5)	C5'—C4'—C8'	121.35 (5)
C4—C5—C6	120.81 (6)	C4'—C5'—C6'	120.19 (5)
C4—C5—C14	121.79 (6)	C4'—C5'—C14'	122.74 (6)
C6—C5—C14	117.38 (6)	C6'—C5'—C14'	116.82 (5)
N5—C6—N1	117.80 (6)	N5'—C6'—N1'	117.96 (6)
N5—C6—C5	124.17 (6)	N5'—C6'—C5'	123.20 (6)
N1—C6—C5	118.01 (6)	N1'—C6'—C5'	118.83 (5)
N3—C7—C3	176.29 (7)	N3'—C7'—C3'	177.41 (7)
C9—C8—C13	119.10 (6)	C9'—C8'—C13'	120.0
C9—C8—C4	120.01 (6)	C9'—C8'—C4'	119.3
C13—C8—C4	120.89 (6)	C13'—C8'—C4'	120.7
C10—C9—C8	120.16 (7)	C8'—C9'—C10'	120.0
C10—C9—H9	119.9	C8'—C9'—H9'	120.0
C8—C9—H9	119.9	C10'—C9'—H9'	120.0
C11—C10—C9	120.37 (7)	C11'—C10'—C9'	120.0
C11—C10—H10	119.8	C11'—C10'—H10'	120.0
C9—C10—H10	119.8	C9'—C10'—H10'	120.0
C12—C11—C10	119.63 (6)	C10'—C11'—C12'	120.0
C12—C11—H11	120.2	C10'—C11'—H11'	120.0
C10—C11—H11	120.2	C12'—C11'—H11'	120.0
C11—C12—C13	120.31 (7)	C13'—C12'—C11'	120.0
C11—C12—H12	119.8	C13'—C12'—H12'	120.0
C13—C12—H12	119.8	C11'—C12'—H12'	120.0
C12—C13—C8	120.42 (6)	C12'—C13'—C8'	120.0
C12—C13—H13	119.8	C12'—C13'—H13'	120.0
C8—C13—H13	119.8	C8'—C13'—H13'	120.0
N4—C14—C5	176.96 (7)	N4'—C14'—C5'	175.70 (7)
C6—N1—C2	124.55 (5)	C6'—N1'—C2'	124.25 (5)
C6—N1—N2	116.97 (5)	C6'—N1'—N2'	116.70 (5)
C2—N1—N2	118.46 (5)	C2'—N1'—N2'	118.98 (5)
N1—N2—H2A	103.6 (8)	N1'—N2'—H2A'	107.0 (8)
N1—N2—H2B	107.6 (8)	N1'—N2'—H2B'	104.9 (9)
H2A—N2—H2B	108.0 (11)	H2A'—N2'—H2B'	110.0 (12)
C6—N5—H5A	117.0 (9)	C6'—N5'—H5A'	120.0 (10)
C6—N5—H5B	119.9 (8)	C6'—N5'—H5B'	121.0 (8)
H5A—N5—H5B	122.0 (12)	H5A'—N5'—H5B'	118.9 (12)
			
O1—C2—C3—C4	−171.43 (7)	O1'—C2'—C3'—C4'	−179.57 (7)
N1—C2—C3—C4	9.53 (10)	N1'—C2'—C3'—C4'	0.40 (10)
O1—C2—C3—C7	6.26 (10)	O1'—C2'—C3'—C7'	1.41 (10)
N1—C2—C3—C7	−172.78 (6)	N1'—C2'—C3'—C7'	−178.62 (6)
C7—C3—C4—C5	179.14 (6)	C7'—C3'—C4'—C5'	175.94 (6)
C2—C3—C4—C5	−3.36 (10)	C2'—C3'—C4'—C5'	−3.01 (10)
C7—C3—C4—C8	−0.34 (10)	C7'—C3'—C4'—C8'	−5.56 (10)
C2—C3—C4—C8	177.16 (6)	C2'—C3'—C4'—C8'	175.49 (6)
C3—C4—C5—C6	−4.87 (10)	C3'—C4'—C5'—C6'	1.73 (10)
C8—C4—C5—C6	174.61 (6)	C8'—C4'—C5'—C6'	−176.75 (5)
C3—C4—C5—C14	176.52 (6)	C3'—C4'—C5'—C14'	175.69 (6)
C8—C4—C5—C14	−3.99 (10)	C8'—C4'—C5'—C14'	−2.79 (10)
C4—C5—C6—N5	−174.96 (6)	C4'—C5'—C6'—N5'	−179.18 (6)
C14—C5—C6—N5	3.70 (10)	C14'—C5'—C6'—N5'	6.51 (10)
C4—C5—C6—N1	6.35 (9)	C4'—C5'—C6'—N1'	2.15 (10)
C14—C5—C6—N1	−174.99 (6)	C14'—C5'—C6'—N1'	−172.16 (6)
C3—C4—C8—C9	50.86 (9)	C3'—C4'—C8'—C9'	−56.14 (7)
C5—C4—C8—C9	−128.61 (7)	C5'—C4'—C8'—C9'	122.33 (6)
C3—C4—C8—C13	−129.66 (7)	C3'—C4'—C8'—C13'	123.55 (6)
C5—C4—C8—C13	50.87 (9)	C5'—C4'—C8'—C13'	−57.99 (7)
C13—C8—C9—C10	0.27 (10)	C13'—C8'—C9'—C10'	0.0
C4—C8—C9—C10	179.76 (6)	C4'—C8'—C9'—C10'	179.7
C8—C9—C10—C11	0.08 (11)	C8'—C9'—C10'—C11'	0.0
C9—C10—C11—C12	−0.60 (12)	C9'—C10'—C11'—C12'	0.0
C10—C11—C12—C13	0.77 (11)	C10'—C11'—C12'—C13'	0.0
C11—C12—C13—C8	−0.43 (11)	C11'—C12'—C13'—C8'	0.0
C9—C8—C13—C12	−0.10 (10)	C9'—C8'—C13'—C12'	0.0
C4—C8—C13—C12	−179.58 (6)	C4'—C8'—C13'—C12'	−179.7
N5—C6—N1—C2	−178.25 (6)	N5'—C6'—N1'—C2'	176.16 (6)
C5—C6—N1—C2	0.53 (10)	C5'—C6'—N1'—C2'	−5.10 (10)
N5—C6—N1—N2	−0.09 (9)	N5'—C6'—N1'—N2'	−0.78 (9)
C5—C6—N1—N2	178.69 (6)	C5'—C6'—N1'—N2'	177.97 (6)
O1—C2—N1—C6	172.72 (7)	O1'—C2'—N1'—C6'	−176.24 (6)
C3—C2—N1—C6	−8.16 (10)	C3'—C2'—N1'—C6'	3.79 (10)
O1—C2—N1—N2	−5.41 (10)	O1'—C2'—N1'—N2'	0.63 (10)
C3—C2—N1—N2	173.70 (6)	C3'—C2'—N1'—N2'	−179.34 (6)

### 1,6-Diamino-2-oxo-4-phenyl-1,2-dihydropyridine-3,5-dicarbonitrile (II) Hydrogen-bond geometry (Å, º)

*Cg*2 is the centroid of the C8–C13 phenyl ring.

**Table:**

*D*—H···*A*	*D*—H	H···*A*	*D*···*A*	*D*—H···*A*
N2—H2*A*···N4^i^	0.913 (13)	2.541 (13)	3.3713 (9)	151.6 (11)
N2—H2*B*···N4^ii^	0.916 (14)	2.495 (14)	3.2404 (10)	138.7 (11)
N2—H2*B*···O1^iii^	0.916 (14)	2.381 (13)	3.0650 (8)	131.5 (11)
N5—H5*A*···N3′^ii^	0.897 (14)	2.525 (14)	3.1431 (9)	126.6 (11)
N5—H5*B*···O1′^ii^	0.930 (14)	1.986 (14)	2.8853 (8)	162.3 (12)
N2′—H2*A*′···O1′^iv^	0.898 (13)	2.186 (13)	3.0608 (8)	164.4 (11)
N2′—H2*B*′···N2^iii^	0.902 (15)	2.681 (14)	3.1829 (8)	116.1 (10)
N2′—H2*B*′···O1	0.902 (15)	2.250 (15)	3.1373 (9)	167.5 (12)
N5′—H5*A*′···N3′^i^	0.887 (15)	2.102 (15)	2.9314 (8)	155.2 (13)
C9′—H9′···*Cg*2	0.95	2.93	3.7972 (5)	153

Symmetry codes: (i) *x*, *y*−1, *z*; (ii) −*x*+1, −*y*+1, −*z*+1; (iii) −*x*+1, −*y*, −*z*+1; (iv) −*x*+2, −*y*, −*z*+1.
